# Rendezvous-assisted endoscopic retrograde pancreatography in groove pancreatitis with a nondilated pancreatic duct

**DOI:** 10.1055/a-2313-4060

**Published:** 2024-05-29

**Authors:** Yawen Liang, Huikai Li, Ke Meng, Baoguo Bu, Yaqi Zhai, Mingyang Li

**Affiliations:** 1 Graduate School of PLA General Hospital, Beijing, China; 2 651943 Division of Gastroenterology and Hepatology, Chinese PLA General Hospital First Medical Center, Beijing, China

A 66-year-old man with a history of alcoholism was referred to our center due to recurrent pancreatitis. Previous surgical exploration with biopsies demonstrated the benign nature of a pancreatic head mass. Enhanced computed tomography (CT) and magnetic resonance cholangiopancreatography (MRCP) revealed a thickened descending duodenum, inflammation in the groove area, and cyst-solid lesion in the pancreatic head, suggesting groove pancreatitis ( Fig. 1 ).

**Fig. 1:**
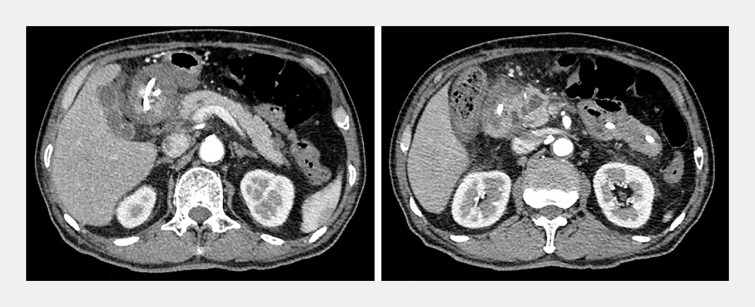
Abdominal contrast-enhanced computed tomography revealed edema and thickening of the duodenal wall, fluid accumulation in the groove area, and enlargement of the pancreatic head with a pseudocyst.

An attempt to place a pancreatic duct stent through endoscopic retrograde pancreatography (ERP) failed due to severe pancreatic duct stricture in the head. Both the guidewire and injection contrast with pressure could not reach the upstream duct, repeatedly passing from the major to minor papilla ( Fig. 2 ).

**Fig. 2:**
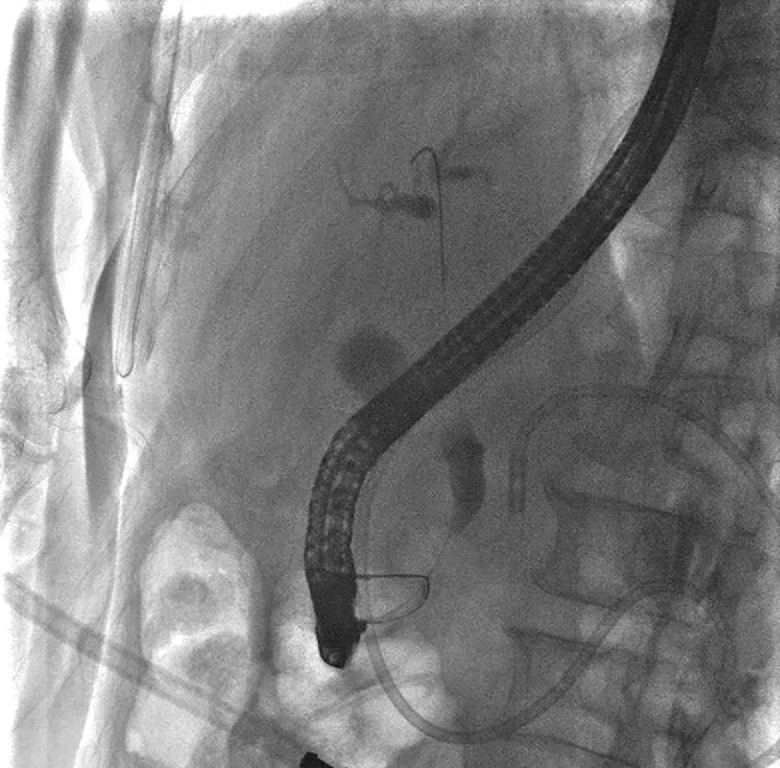
The guidewire passed from the major papilla to minor papilla.

Then rendezvous-assisted ERP (RV-ERP) was planned. Endoscopic ultrasound (EUS) revealed a non-dilated pancreatic duct (2.6 mm) in the body. A 19-G needle was used to puncture the main duct through the stomach, and the contrast injection confirmed the pancreatic duct stricture ( Fig. 3 ). After several attempts, the guidewire was finally advanced into the duodenum ( Fig. 4 ). Then the echoendoscope was exchanged for a duodenoscope, and the guidewire was retrieved with a snare. A sphincterotome was inserted over the rendezvous guidewire for dilation and sphincterotomy. Finally, a 7-Fr × 9-cm pancreatic plastic stent (Zimmon; Cook, Limerick, Ireland) was successfully placed for ductal dilation and decompression ( Video 1 , Fig. 5 ).

**Fig. 3:**
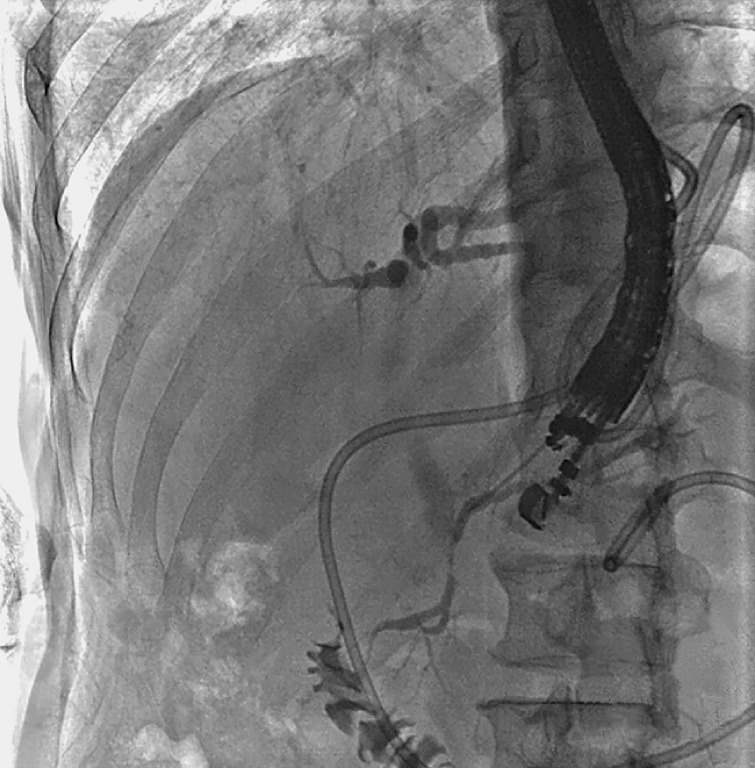
The puncture needle penetrated the pancreatic duct.

**Fig. 4:**
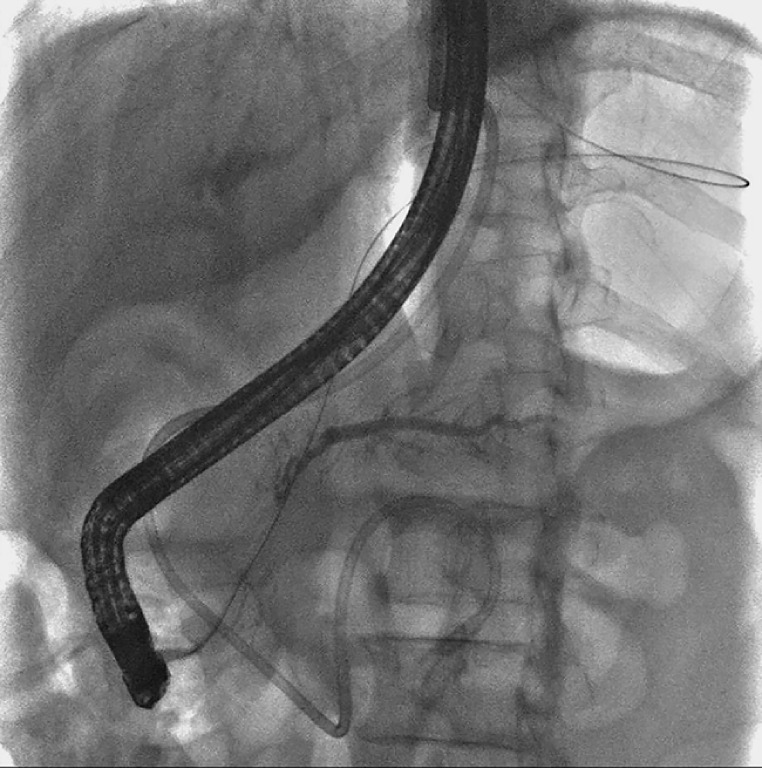
The guidewire passed from the main pancreatic duct into the duodenum.

**Fig. 5:**
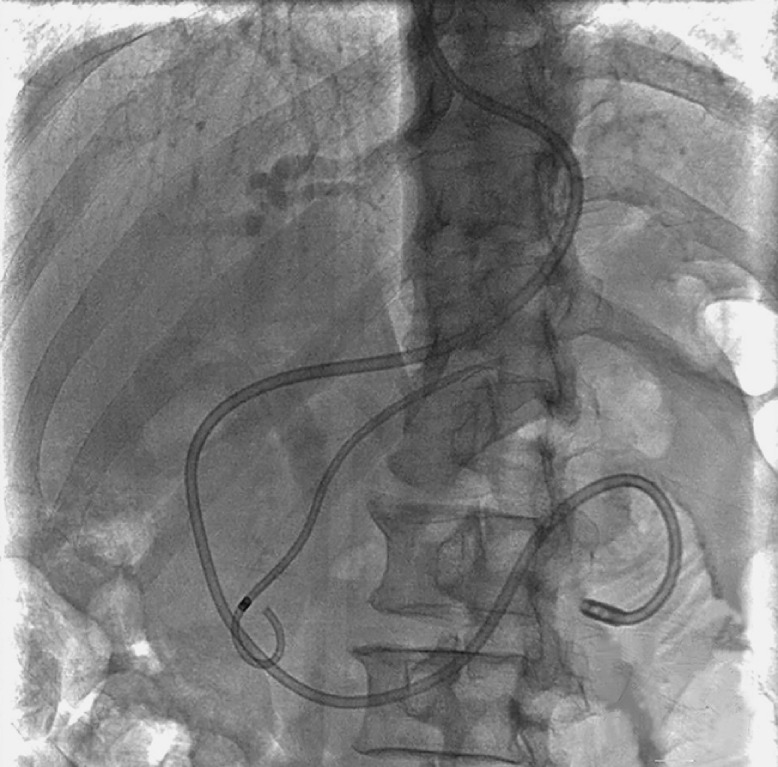
Successful placement of a stent.

Rendezvous-assisted endoscopic retrograde pancreatography in groove pancreatitis with a nondilated pancreatic duct.Video 1

Groove pancreatitis is a rare form of chronic pancreatitis that affects the paraduodenal area 1 . Currently, its treatment strategy remains controversial, and endoscopic treatment is often indicated for patients with chronic pain not responding to conservative treatment 2 . When conventional ERP fails, RV-ERP could be an effective alternative method 3 . In our case, RV-ERP is also feasible even for patients with nondilated pancreatic ducts. To our best knowledge, this is also the first case of RV-ERP for the treatment of groove pancreatitis.

Endoscopy_UCTN_Code_TTT_1AS_2AD

## Notes

Endoscopy E-Videos https://eref.thieme.de/e-videos
*E-Videos* is an open access online section of the journal *Endoscopy* , reporting on interesting cases and new techniques in gastroenterological endoscopy. All papers include a high-quality video and are published with a Creative Commons CC-BY license. Endoscopy E-Videos qualify for HINARI discounts and waivers and eligibility is automatically checked during the submission process. We grant 100% waivers to articles whose corresponding authors are based in Group A countries and 50% waivers to those who are based in Group B countries as classified by Research4Life (see: https://www.research4life.org/access/eligibility/ ). This section has its own submission website at https://mc.manuscriptcentral.com/e-videos .

## References

[1] Patel BN Jeffrey RB Olcott EW Groove pancreatitis: a clinical and imaging overview Abdom Radiol (NY) 2020 45 1439 1446 31559471 10.1007/s00261-019-02239-1

[2] Ukegjini K Steffen T Tarantino I Systematic review on groove pancreatitis: management of a rare disease BJS Open 2023 7 zrad094 10.1093/bjsopen/zrad094 37749756 PMC10519812

[3] Ergun M Aouattah T Gillain C Endoscopic ultrasound-guided transluminal drainage of pancreatic duct obstruction: long-term outcome Endoscopy 2011 43 518 525 10.1055/s-0030-1256333 21437853

